# Transradial peripheral vascular intervention using Fowler’s position and Terumo R2P system for patients with heart failure: two case reports

**DOI:** 10.1186/s13256-021-03242-1

**Published:** 2022-01-21

**Authors:** Akihiro Nakamura, Kenjiro Sato, Hideaki Endo

**Affiliations:** Department of Cardiology, Iwate Prefectural Central Hospital, 1-4-1, Ueda, Morioka, Iwate 020-0066 Japan

**Keywords:** Fowler’s position, Peripheral artery disease, R2P, Transradial intervention, Vac-Lok

## Abstract

**Background:**

Positioning a patient on the catheterization table is important for proper cardiac or respiratory function during peripheral vascular interventions. Fowler’s position, where the patient’s head is a 45° angle, is more effective in reducing venous blood volume returning to the heart from the periphery compared with the supine position. The Terumo R2P system has been developed for transradial peripheral vascular interventions.

**Case presentation:**

Two patients with heart failure (a 75-year-old Japanese female and a 74-year-old Japanese male) underwent lower-extremity peripheral vascular interventions in Fowler’s position to prevent worsening heart failure. Because their head position was opposite the C-arm of the X-ray machine, the left radial artery was selected as the access site. The Terumo R2P system was used for transradial peripheral vascular intervention. We successfully treated superficial artery diseases with long shaft balloons and rapid-exchange Terumo R2P Misago stents.

**Conclusions:**

Although lower-extremity peripheral vascular intervention using Fowler’s position and the Terumo R2P system has several limitations, including device availability and technical complexity, it may be effective for particular patients who have higher risk of worsening heart failure in the supine position.

## Introduction

Fowler’s position, in which the patient’s head is placed at a 45° angle to the table, is often used to accommodate patients comfortably [1]. This position reduces central fluid volume due to decreased venous return from the periphery, which prevents worsening heart failure (HF) [2]. Although peripheral vascular interventions (PVIs) are typically done in the supine position, Fowler’s position can be helpful for HF patients with peripheral arterial disease (PAD) because it decreases the ventricular preload. Herein, we report two PAD cases with HF who underwent lower extremity PVI using Fowler’s position and the Terumo R2P system, which has been developed for transradial PVI [3].

## Case presentation

**Fig. 1:**
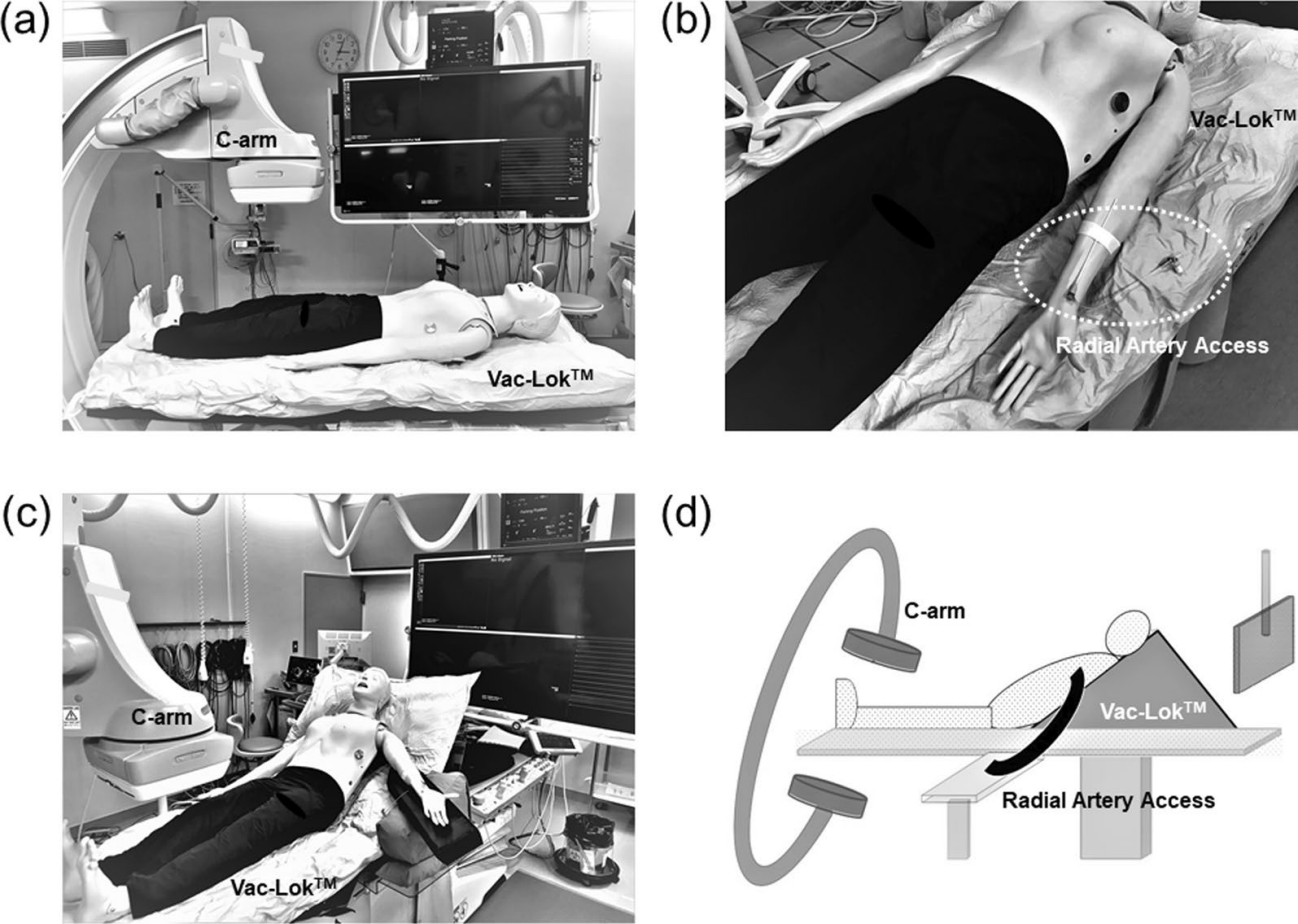
**a** Patient lying on the Vac-Lok device in the opposite direction of the C-arm on the X-ray machine. **b** Left radial artery as the vessel access site. **c** Patient’s upper body fixed in Fowler’s position. **d** Schema showing the patient in Fowler’s position on the catheterization table during transradial peripheral vascular intervention

Each patient was initially put in the supine position on the catheterization table with a Vac-Lok fixation device (CIVCO Medical Solutions, Orange City, IA), opposite from the C-arm of the X-ray machine (Fig. 1a) [4]. As shown in Fig. 1b, the left radial artery (RA) was selected as the vessel access site with the R2P system (Terumo, Tokyo, Japan). This device has been developed and specialized for treatment of lower-extremity PAD via radial access [3]. A sheath was inserted into the left RA, either a 7 Fr 16-cm long R2P Glidesheath^®^ slender sheath (Terumo, Tokyo, Japan) or a 6 Fr 119- or 149-cm long R2P Destination slender sheath (Terumo, Tokyo, Japan). The 7 Fr R2P Glidesheath slender sheath has a thin-walled layer, and the outer diameter is the same size as that of current 6 Fr sheaths [3]. To position the 7 Fr R2P Glidesheath sheath, a 7 Fr 120-cm long R2P SlenGuide guiding catheter (Terumo, Tokyo, Japan) needs to be inserted into the sheath and advanced to the right or left iliac artery using a 0.035 inch Rafifocus stiff J-shaped 380-cm long guidewire (Terumo, Tokyo, Japan). This guiding catheter also has a thin-walled design and thin inner lumen to accommodate balloons or stents [3]. The 6 Fr R2P Destination slender sheath is relatively new. It has been developed for radial access, and it is specifically designed to have greater flexibility and tracking [5]. The 6 Fr R2P Destination slender sheath needs to be directly advanced into the right or left iliac artery, if possible, and into the femoral artery with a 0.035 inch Rafifocus stiff long guidewire. After the vascular access procedure was completed, the patient’s upper body was fixed at a 45° angle (so-called Fowler’s position) with a Vac-Lok fixation device (Fig. 1c, d).

### Case 1

**Fig. 2:**
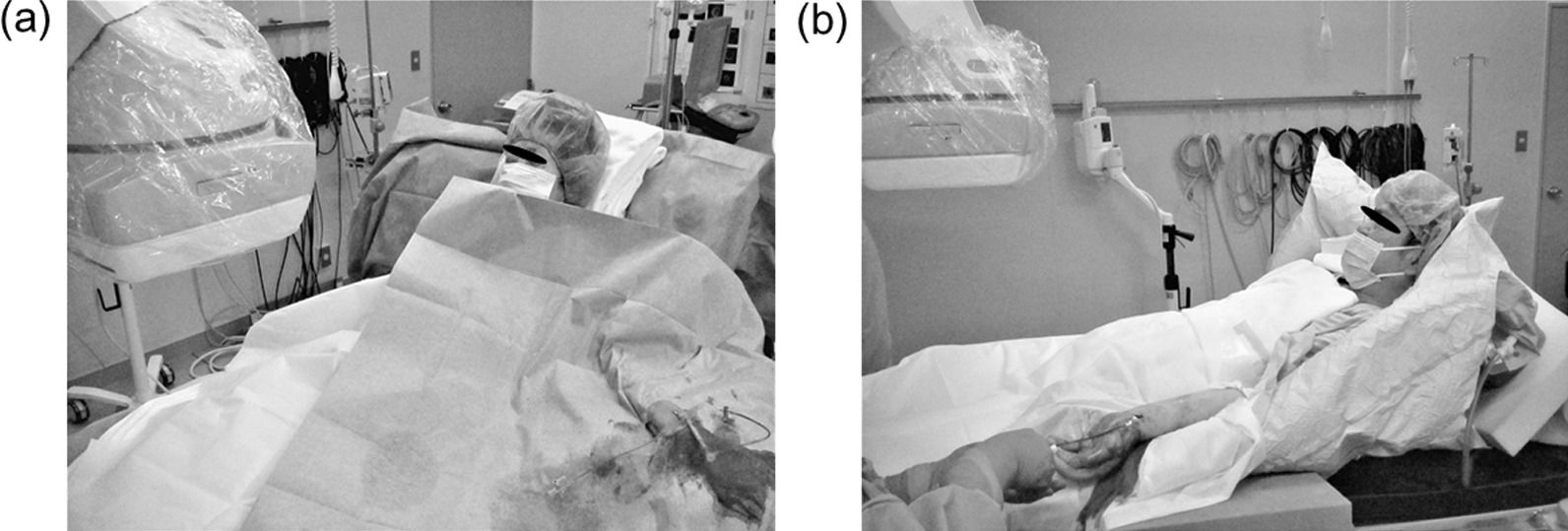
Case 1 patient **a** and case 2 patient **b** in Fowler’s position during transradial peripheral vascular intervention

**Fig. 3:**
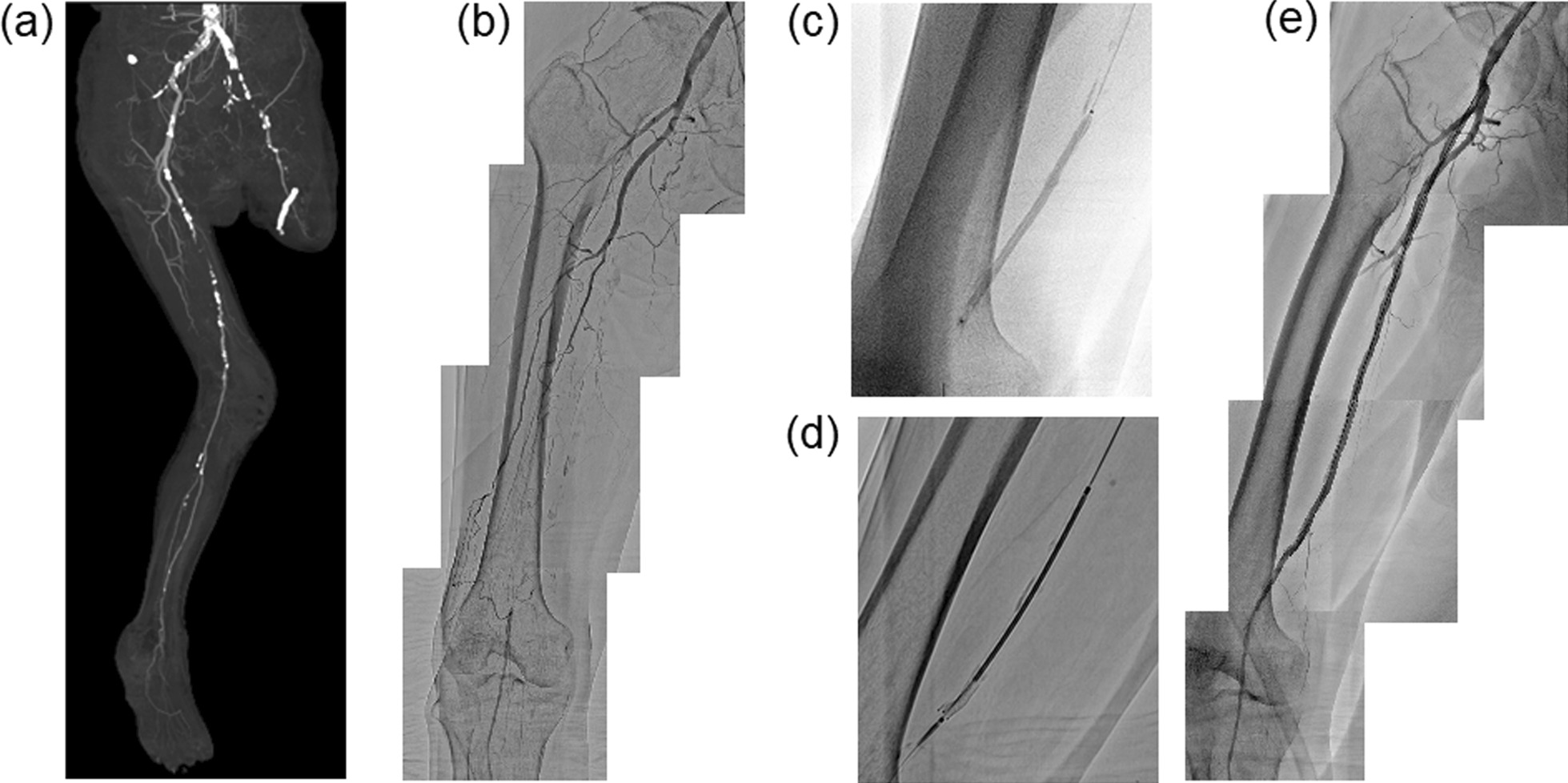
Computed tomography (CT) angiographic image showing total occlusion of the left superficial femoral artery (SFA) and the left iliac artery (**a**). Angiography of the right SFA before and after peripheral vascular intervention (PVI), shown in **b**–**e**. Initial angiography showing total occlusion of the left SFA from the ostium to the distal portion (**b**). PVI was performed with balloon inflation **c** and stent implantation (**d**). Final angiography after PVI showing a good result (**e**)

A 75-year-old Japanese female (height: 150 cm; weight: 43 kg) was emergently admitted to our hospital for worsening HF due to ischemic cardiomyopathy, with a left ventricular ejection fraction less than 35%. The patient had a medical history of diabetes mellitus and smoking. She had been diagnosed with PAD at 73 years of age, and she underwent amputation surgery above the left knee owing to ischemic and diabetic necrosis 1 year prior. Although her symptoms for HF had improved after resting in Fowler’s position and treatment with diuretics, beta blockers, and angiotensin-converting enzyme inhibitors, she complained of rest pain in her right lower leg 1 week after admission. Physical findings showed absent pedal pulses, and shiny smooth, pallor, cold skin of the right leg. The ankle-brachial index (ABI) was unmeasurable at rest. A computed tomography (CT) angiogram showed chronic total occlusion (CTO) of the right superficial femoral artery (SFA) and left external iliac artery (Fig. 3a). She was diagnosed as critical limb ischemia (clinical limb stage: Fontaine III). We decided to perform PVI for revascularization of the right SFA during this hospitalization because of severe pain uncontrolled by pain relief medicines. We considered that she would still need to be in Fowler’s position on the catheterization table during PVI to prevent worsening HF. After insertion of a 7 Fr R2P Glidesheath slender sheath and advancement of a 7 Fr 120-cm long SlenGuide catheter into the right iliac artery, the patient was fixed in Fowler’s position with the Vac-Lok fixation device (Fig. 2a). Initial angiography showed total occlusion of the left SFA from the ostium to the distal portion (Fig. 3b). We advanced a 0.014 inch Halberd guidewire (Asahi Intecc, Nagoya, Japan) with a 2.5 Fr 150-cm long Corsair Armet microcatheter (Asahi Intecc, Nagoya, Japan) into the right SFA. We successfully crossed the wire into the CTO lesion of the right SFA, and we dilated the lesion with a 4.0–100 mm Metacross rapid-exchange balloon catheter (Terumo, Tokyo, Japan) (Fig. 3c). After predilation, we implanted two rapid-exchange 6.0–100 mm R2P Misago stents (Terumo, Tokyo, Japan) with a 200-cm long shaft system in the CTO lesion of the right SFA (Fig. 3d). Following postdilation of the stents with a 5.0–100 mm Metacross balloon catheter, we confirmed favorable blood flow below the right knee without residual stenosis and dissection (Fig. 3e). The guiding sheath in the right iliac artery and the sheath in the left RA were withdrawn and the puncture site was managed via continuous compression using TR Band (Terumo, Tokyo, Japan), a hemostasis device used for transradial catheterization. We did not use vasodilators, and we had no access site-related complications. HF did not worsen during PVI nor in the postoperative period. The ABI had improved to 0.78 in the right leg, and she was discharged 1 week after her PVI. At 6 months after PVI, she was completely free from pain in her right lower leg. ABI at rest of the leg maintained a relatively good level (0.72) and chronic HF had been well managed medically.

### Case 2

**Fig. 4:**
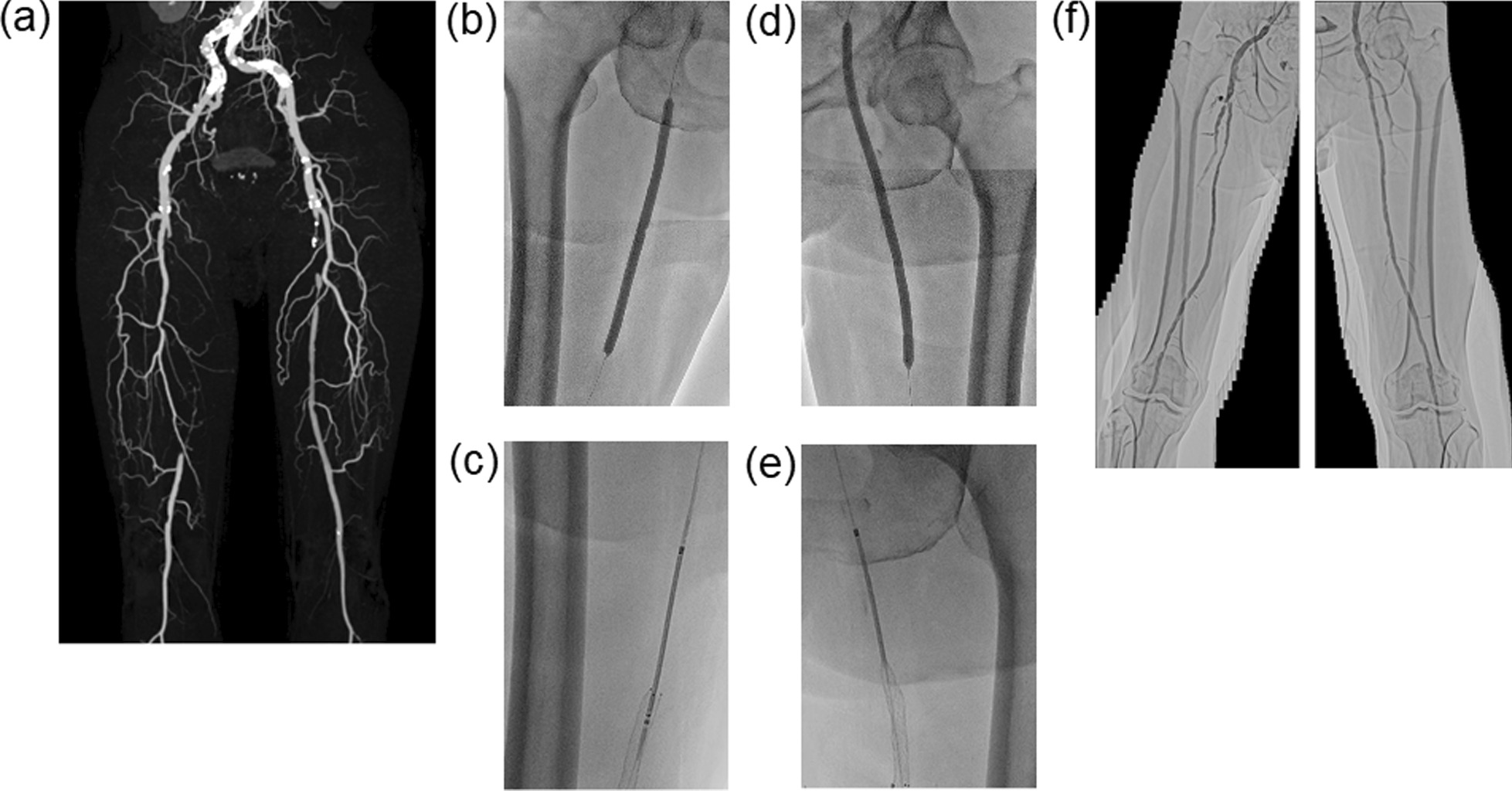
**a** Computed tomography (CT) angiographic image showing total occlusion of the right and left superficial femoral artery (SFA): from the ostium to the distal portion in the right SFA; from the ostium to the mid portion in the left SFA. Angiography of the right and left SFA before and after peripheral vascular intervention (PVI), shown in b–f. PVI in the right SFA was performed with balloon inflation **b** and stent implantation (**c**), and that in the left SFA was performed with balloon inflation **d** and stent implantation (**e**). Final angiography after PVI showing good results in both legs (**f**)

A 74-year-old Japanese male (height: 161 cm; weight: 56 kg) with a medical history of hypertension, dyslipidemia, and smoking was admitted to our hospital for treatment of PAD with a 5-month history of intermittent claudication (Fontaine classification: IIb) in both his lower legs, despite optimal medication. Physical findings showed diminished pedal pulses and cold skin of both legs. The patient’s maximum walking distance was about 150 m, and the right and left leg ABI scores at rest were 0.52 and 0.64 with monophasic wave forms, respectively. A CT angiogram showed CTO lesions in the proximal portion of both SFAs (Fig. 4a). Although we decided to intervene on CTO lesions of both SFAs, there were concerns his HF could get worse during PVI. He had a history of two hospitalizations due to hypertensive HF and a worsening HF event during cardiac catheterization. Thus, the patient underwent PVI procedures for SFA CTO lesions on both sides. After successful insertion of a 7 Fr Glidesheath slender sheath into the left RA, the patient was put in Fowler’s position using the Vac-Lok fixation device (Fig. 2b). We advanced a 7 Fr 120-cm-long SlenGuide catheter into the right iliac artery, and we tried to advance a 0.014-inch Gladius guidewire (Asahi Intecc, Nagoya, Japan) into the right SFA. Unfortunately, we failed to pass the wire through the CTO lesion in the right SFA due to inadequate backup support by the guiding catheter. To strengthen the backup force during advancement, we removed the guiding catheter from the sheath and exchanged the sheath for a 6 Fr 119-cm long R2P Destination slender sheath. The Destination slender sheath was advanced into the right external iliac artery, and then a 0.014-inch Gladius guidewire (Asahi Intecc, Nagoya, Japan) with a 2.8 Fr 150-cm long Corsair PV microcatheter (Asahi Intec, Nagoya, Japan) was advanced into the right SFA. We managed to cross the wire through the CTO in the right SFA, and we dilated the lesion with a 6.0–200 mm Crosstella rapid-exchange balloon catheter (Terumo, Tokyo, Japan) (Fig. 4b). After predilation, we implanted three R2P Misago rapid-exchange stents; 6.0–100, 60, and 40 mm, using a 200-cm long shaft system in the CTO lesion in the right SFA (Fig. 4c). Postdilation was done with the same balloon as predilation. Next, we performed endovascular treatment (EVT) for the CTO lesion in the left SFA. We advanced a Destination slender sheath into the left external iliac artery and crossed a 0.014-inch Gladius guidewire with a Corsair PV microcatheter through the left SFA. After predilation with a 6.0–200 mm Crosstella balloon catheter (Fig. 4d), a 7.0–100 mm R2P Misago stent was implanted (Fig. 4e). Postdilation was done with the same balloon as predilation. The final angiography showed no residual stenosis, no dissection, and favorable blood flow in both legs (Fig. 4f). TR Band was used to achieve hemostasis after the sheath was removed from the left RA. We had no access site-related complications. He showed no worsening HF and complained of no intermittent claudication after the procedure. His ABI improved to 1.00 in the right leg and 1.01 in the left leg. He was discharged 2 days after his PVI. At 12 months after PVI, he was completely free from quality of life-threatening claudication, with good levels of ABI at rest (right: 0.95; left: 0.98) and chronic HF had been well-managed medically.

## Discussion

We present two cases of PAD patients with HF who underwent transradial endovascular intervention. The patients were placed in Fowler’s position on the catheterization table during the PVI procedure in order to avoid worsening congestive HF.

A patient’s position plays a vital role in the management of HF, especially to prevent worsening HF [6]. During percutaneous coronary or peripheral intervention, patients are usually put in the supine position on the catheterization table. In this position, venous blood in the abdomen and lower extremities is mobilized, and venous return to the right ventricle increases, further elevating pulmonary venous and capillary pressure. This may result in a patient perceiving dyspnea due to pulmonary edema, with a decrease in pulmonary compliance and an increase in airway resistance [2, 7, 8]. Compared with the supine position, a semi-sitting position can decrease venous return and is more comfortable for patients with HF [7, 8]. Thus, we put patients in Fowler’s position to prevent worsening HF in the intra- and perioperative period.

In our case, the RA was selected as the access site because the patients were positioned with their head and legs reversed on the table. A brachial approach was not considered suitable because it has been linked with higher risk of large hematoma and pseudoaneurysm formation compared with the RA approach [9]. In our case, the Terumo R2P system, which has been developed for transradial peripheral intervention, was used [3]. As shown in case 2, the R2P system with Destination slender sheath seems to have a more powerful back force, compared with the Glidesheath slender sheath and SlenGuide> catheter, in the treatment of long and complex SFA lesions. Although the safety of this system in transradial coronary or peripheral intervention has already been reported [3, 10], Nazir *et al.* recently reported a case of severe RA spasm, causing entrapment of the Destination slender sheath and requiring emergency surgery for sheath removal [5]. We believe it would be meaningful to develop sheaths smaller in diameter to perform treatment in a less invasive manner; however, at the same time, we need to be aware of potentially serious complications such as vessel hematoma, dissection, major bleeding, and refractory spasms [10].

Body position affects chest wall volume, lung function, and respiratory muscle strength. Spirometric parameters in healthy young patients were reported to be better in Fowler’s position than in the supine position [11]. Although further studies are necessary, Fowler’s position may also be more comfortable for patients without HF.

## Conclusion

To the best of our knowledge, this is the first report on PVI procedures conducted while patients with HF were in Fowler’s position. The left RA approach, implementing the Terumo R2P system was useful for those lying down on the catheterization table in the opposite direction of the X-ray machine. Although device availability limits the RA approach, we believe this is a viable method, at least in patients at risk of worsening HF.

## Data Availability

The data used in the case report are available on reasonable request.

## Abbreviations

HF: Heart failure
PVI: Peripheral vascular interventions
PAD: Peripheral arterial disease
RA: Radial artery
ABI: Ankle-brachial index
CT: Computed tomography
CTO: Chronic total occlusion
SFA: Superficial femoral artery
